# Prevention of hip fractures in older adults residing in long-term care facilities with a hip airbag: a retrospective pilot study

**DOI:** 10.1186/s12877-022-03221-1

**Published:** 2022-06-30

**Authors:** Banne Nemeth, Marleen van der Kaaij, Rob Nelissen, Jan-Kees van Wijnen, Katja Drost, Gerard Jan Blauw

**Affiliations:** 1 Department of Clinical Epidemiology, Leiden University Medical Center, Albinusdreef 2, 2333ZA Leiden, The Netherlands; 2 Department of Orthopedic Surgery, Leiden University Medical Center, Albinusdreef 2, 2333ZA Leiden, The Netherlands; 3 Department of Internal Medicine, Geriatrics, Amstelland Ziekenhuis, Amstelveen, The Netherlands; 4 tanteLouise, Bergen op Zoom, The Netherlands; 5 Department of Internal Medicine, Geriatrics, Leiden University Medical Center, Leiden, The Netherlands

**Keywords:** Hip airbag, Hip fracture, Falling, Prevention, Technology

## Abstract

**Background:**

Hip and pelvic fractures do commonly occur among older adults. This pilot study aimed to evaluate the effect of introduction of the WOLK hip airbag on the incidence of hip fractures.

**Methods:**

A retrospective study was performed among 969 participants residing within 11 long-term care facilities for older patients, belonging to one large healthcare organization in The Netherlands. The intervention concerned application of 45 WOLK hip-airbags, distributed among selected residents of the long-term care facilities. Inclusion criteria; physically active participants with a pelvic circumference between 90-125 cm able to wear the hip airbag. Exclusion criteria; participants who continuously removed the hip airbag themselves or participants who depended on a wheelchair for mobility. Main outcome measures were the occurrence of falls and hip, pelvic and other fractures.

**Results:**

The incidence of hip and pelvic fractures declined from 3.3/100 person years to 1.8/100 person years during the study for an Incidence Rate Ratio (IRR) of 0.55 (95% confidence interval (95%CI) 0.34–0.87) in the entire study population. The incidence of other fractures did not decline during the study period (IRR 0.72;95%CI 0.37–1.40). The incidence of falls declined to some extent during the study (IRR 0.88; 95%CI 0.83–0.93).

**Conclusions:**

After introduction of the WOLK hip airbag a reduction of the incidence of hip and pelvic fractures by almost half was observed in older patients residing in long-term care facilities, even though only 45 hip airbags were distributed among the 969 residents. As selection bias cannot be ruled out in this study, the results of this pilot study warrant replication by a future clinical trial to determine true effectiveness of this intervention.

## Introduction

Falls and hip and pelvic fractures are an common problems amongst older patients, affecting millions worldwide each year. An estimated one third of all patients aged >  = 65 years fall each year, and half of those fall repeatedly [1] . Around 10% of those falls result in hospital admissions, of which 50% are due to hip fractures [2–6]. Taking into account that the population is aging globally, this leads to a massive burden on health resources. Hip and pelvic fractures are associated with negative outcomes for patients such as mortality, functional impairment, loss of independence and reduced quality of life [7–11] . Despite awareness, multiple interventions to reduce fall risk but especially interventions to reduce hip fracture risk in frequent fallers have not been successful [12].

Hip protectors are one such intervention, and have been around since the late 1980s. Traditionally, this are either hard or soft protectors worn in specially designed underwear over the trochanter major region, and meant to shift the energy of the fall away from the trochanter major, distally along the shaft of the femur. The latest Cochrane review by Santesso et al. showed that among patients in an institutional setting a small reduction in hip fractures might be achieved with application of hip protectors (risk ratio (RR) 0.82, 95% confidence interval (CI) 0.67 to 1.00), however the result is not statistically significant, even though 14 trials with 1108 participants were pooled [13]. The reasons for this limited success is thought to be multifactorial. First, the efficacy of a soft/hard protector with padding may not be optimal. Additionally, its limited success could at least partly be related to barriers to hip protector use, such as limited acceptance and poor adherence. In other words, if the hip protector is not worn, it does not offer any protection [14]. A recent study demonstrated a three-fold reduction in hip fractures among long-term care residents if a hip protector was worn at the time of the fall, [15] thus proving that hip protectors could potentially work well to reduce hip and pelvic fractures. Contrary, a key trial showed no effectiveness in individuals wearing the hip protector at the time of a fall, although some limitations did also apply to this trial [16].

The WOLK hip airbag is a hi-tech innovation that is comfortable and easy to wear, especially compared to hard and ‘bulky’ hip protectors. It aims to protect older adults against hip and pelvic fractures and has been developed in cooperation with Delft University of technology in The Netherlands. It is comprised of a belt with two airbags on each side for the left and right hip that can easily be worn underneath any type of clothing. The airbag (over the trochanter major region) inflates once a fall is detected and before the hip region makes contact with the floor.

This pilot study aimed to evaluate the effect of introduction of the WOLK hip airbag on the incidence of hip and pelvic fractures of residents in long-term care facilities (residential care).

## Methods

### Study design and population

In this retrospective study, healthcare data from older adults residing in long-term care facilities (belonging to a large healthcare organization in The Netherlands) were collected. Participants were permanent residents of long-term care facilities, they were often functionally dependent in their activities of daily living and needed support, supervision and care around the clock. Their reason for admission was a psychogeriatric diagnosis, i.e. dementia, in an advanced stage. In addition, multiple comorbidities such as heart failure, chronic obstructive pulmonary disease or Diabetes Mellitus were often present. All long-term care facilities accommodated patients entitled to care package ZZP5 to ZZP7 (‘living with intensive care’ [ZZP5] to ‘living with highly intensive care [with psychiatric disorder/dementia]’ [ZZP7]).

### The hip airbag

**Fig. 1:**
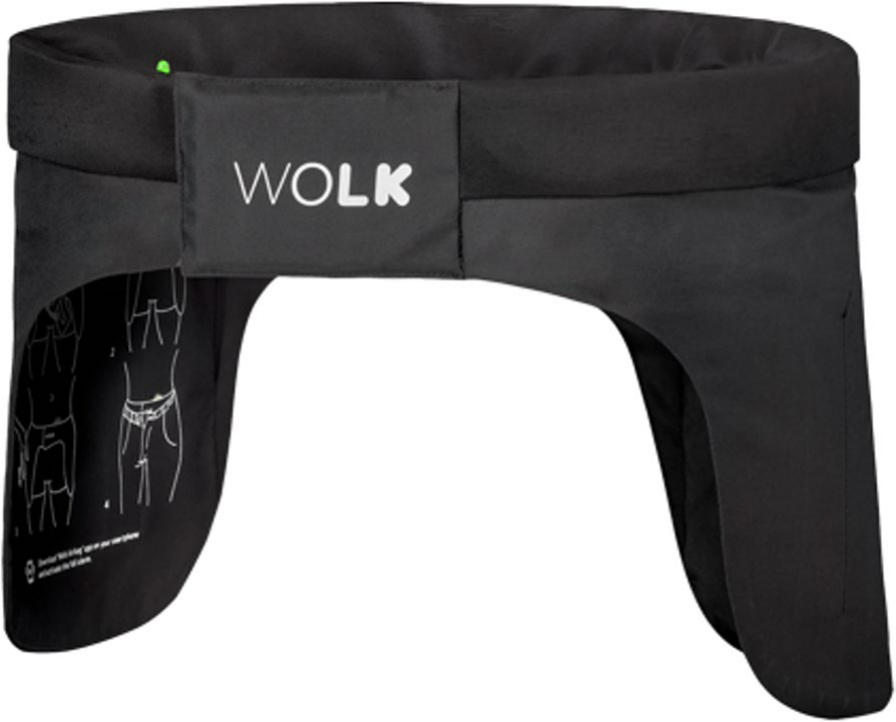
The WOLK hip airbag

The WOLK hip airbag is shown in Fig. 1. It contains three inertial measurement units (sensors) which measure the wearer’s movements and position in relation to the floor. A central computer within the device analyses directional and acceleration data and automatically detects if a fall occurs. When an individual falls, an inflating device with a small carbon dioxide container inflates the hip airbag on either the left or right side of the wearer. After deployment, it can be worn again by replacing the carbon dioxide container. The impact reduced capacity of the WOLK has been extensively tested in a biomechanical laboratory. The inflation process takes less than 100 ms. Automatic fall detection is thus included and data on falls and airbag activation are transmitted by GSM signal (if this was preferred by the wearer). The hip airbag is machine washable. The WOLK airbag has been CE approved and as such has been extensively tested on safety.

### Study intervention

**Fig. 2:**
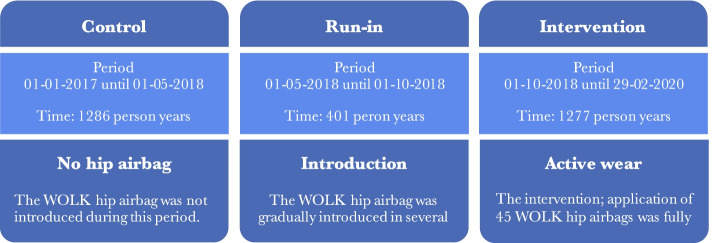
Study period overview

This retrospective study is a before- after comparison of the introduction of the WOLK hip airbag in 11 residential care homes belonging to one long-term care organization in The Netherlands. Figure 2 shows the study design and study periods. In the first period (control period), the WOLK hip airbag was not yet introduced. During the run-in period, 45 WOLK hip airbags were gradually introduced among residents, each facility had multiple hip airbags available. The intervention period started once all 45 WOLK hip airbags were in use. Only 45 hip airbags were used as this was a pilot study. The WOLK hip airbag was introduced as part of a quality improvement initiative, initiated by a long-term care facility in The Netherlands. The number of hip airbags was limited due to costs and due to the fact this was a pilot study (and at the time of study initiation, a promising but unproven medical device). The WOLK airbag was given to individuals with wandering behaviour (a high risk of repeated falls in whom other methods to reduce falling were deemed unsuccessful, as assessed by the physician, physiotherapist and nursing staff). In addition, the following practical inclusion criteria were used; patients needed to have a pelvic circumference between 90-125 cm (measured 1 hand width beneath the umbilicus), sufficient mobility to be able to at least transfer from bed or chair to a standing position using their own muscle power and permission from the physician and the patient representative for inclusion. The WOLK hip airbag was not given to participants who continuously removed the hip airbag themselves or to participants who depended on a wheelchair for mobility (without the ability to get out themselves). If a hip airbag was applied, the device was usually worn during daytime. For individuals with a high fall risk at night, the hip airbag was worn for 24 h. Most selected wearers were patients who had been instructed not to stand up and start walking without supervision because of a high risk of falling, but were unable to follow these instructions due to memory deficits caused by i.e. dementia. The number of residents eligible to wear the hip airbag outnumbered the number of available hip airbagsdue to reasons described earlier (costs and pilot study). The hip airbag was not worn by the same individual every day.

### Data collection

Data on falls, hip, pelvic and other fractures of residents were collected from electronic incidence reports for all residents. Falls and fractures occurring between 01–01-2017 and 29–02-2020 were included in the study, data collection stopped before COVID-19 infections occurred in The Netherlands. Demographic data were electronically extracted from the electronic patient records and summarized for the median of the study periods, so the first half of 2018, the second half of 2018 and the first half of 2019. All data were collected on a population level, this means that individual patient data were not available.

### Statistical analyses

Demographic data were summarized as means or proportion as appropriate. The Incidence Rate (IR) including 95% Confidence Interval (95%CI) of falls and hip, pelvic and other fractures was calculated over three different study periods. The IR was therefore estimated by dividing the number of falls or fractures by the number of person years. The number of person years was calculated by the number of person days/365.25. The number of person days was estimated by multiplying the number of residents by the number of days within the study period (assuming an open dynamic cohort). This approach marginally overestimated the total person-time (and thus underestimated incidence rates) as for instance, patients admitted to the hospital were still considered “at risk” during the hospitalized period. Second, the Incidence Rate Ratio (IRR) was calculated to compare the incidence of falls or fractures between study periods.

### Ethics approval

In this study, anonymous patient data were summarized and analysed following introduction of the hip airbag. Ethics committee approval was therefore by Dutch law, deemed unnecessary according to national legislation (Wet medisch-wetenschappelijk onderzoek met mensen, 26 februari 1998). Patients (or their legal representatives) provided informed consent to use the hip airbag, this was register in a patient’s personal electronic patient file. All methods were carried out in accordance with relevant guidelines and regulations.

## Results

### Study population and period

Over three study periods, the number of participants gradually declined (from 969 in the control period to 904 in the intervention period), shown in Table 1, constituting to a total of 2.964 person years. 70% were females and 30% males. The composition of the population remained stable in terms of indications for admission during the study period.

**Table 1:** Study period details

	**Control**	**Run-in**	**Intervention**
Start date	01–01-17	01–05-18	01–10-18
End date	01–05-18	01–10-18	29–02-20
Study days, n	485	153	516
Patients, n	969	958	904
Person years, n	1286	401	1277
Sex			
Female, %	70	70	69

### Device compliance

Device compliance (to what extent participants accepted the hip airbag) was good, some participants needed a few days to get used to the device as part of their daily routine. Others with an advanced stage of dementia occasionally removed the hip airbag themselves, though this could be handled by nursing staff. All participants in this study wear compliant, meaning they did tolerate wearing the hip airbag during the intervention period.

### Falls and fractures

Table 2 shows that a total of 72 hip and pelvic fractures and 35 other fractures occurred during the complete study period among all individuals (total number during the complete study period), for an Incidence rate (IR) of 2.5/100 person years (py) (95% CI 2.0 to 3.2) and IR of 1.2/100py (95%CI 0.9 to 1.7) respectively. Besides hip fractures (*n* = 64) and pelvic fractures (*n* = 8), humeral, forearm and lower-leg fractures (*n* = 8 for each of these) were most common.

**Table 2:** fractures per study phase

	**Control**	**Run-in**	**Intervention**
**Falls**			
Number of falls	2719	798	2067^a^
**Hip and pelvic fractures**			
Hip, n	37	6	21
Pelvis, n	5	1	2
	**42**	**7**	**23**
**Other fractures**			
Femur^b^	1	0	1
Humeral	4	2	2
Spine	3	0	0
Finger	1	0	0
Skull	0	0	1
Knee	1	0	0
Nose	0	0	1
Forearm	4	1	3
Lower-leg (including ankle)	4	1	3
Clavicle	0	1	1
	**18**	**5**	**12**

^a^Number of falls restricted until 01–01-2020 due to availability of fall data until this date

^b^Excluding hip or any (sub)trochanteric fracture types

### Effectiveness

The occurrence of falls declined to some extent across all study periods, with an IR of 2.1/100py, 2.0/100py and 1.8/100py for the control, run-in and intervention period, respectively, as shown in Table 3. The incidence of hip fractures declined from 2.9/100py to 1.6/100py during the study, for an Incidence Rate Ratio (IRR) of 0.56 (95%CI 0.34–0.92) (IRR shown for a comparison between the run-in and intervention period combined versus the control period). An equal result was found for pelvic fractures, although these fractures occurred much less common (IRR 0.46 (95%CI 0.11–1.92). The incidence of other fractures such as humeral, forearm, skull, ankle or clavicular fractures did not decline over time, for an IRR of 0.72 (95%CI 0.37–1.40).

**Table 3:** Incidence rates and ratio

	**Control**	**Run-in**	**Intervention**	**Effectiveness**
		**Incidence rate/py (95%CI)**		**Incidence rate ratio (95%CI)**
Falls	2.1 (2.0—2.2)	2.0 (1.9–2.1)	1.8 (1.7–1.9)^a^	0.88 (0.83–0.93)
		**Incidence rate/100py (95%CI)**		**Incidence rate ratio (95%CI)**
Hip fractures	2.9 (2.1–3.9)	1.5 (0.6–3.1)	1.6 (1.0–2.5)	0.56 (0.34–0.92)
Pelvic fractures	0.4 (0.2–0.9)	0.2 (0.02–1.2)	0.2 (0.03–0.05)	0.46 (0.11–1.92)
Hip + pelvic fractures	3.3 (2.4–4.4)	1.7 (0.8–3.4)	1.8 (1.2–2.7)	0.55 (0.34–0.87)
Other fractures	1.4 (0.9–2.2)	1.2 (0.5–2.7)	0.9 (0.5–1.6)	0.72 (0.37–1.40)

IRR for the Intervention and Run-in study period in comparison to the control period

^a^restricted up to 01–01-2020, due to availability of fall data until this date

## Discussion

In this retrospective study comparing the incidence of hip and pelvic fractures before and after introduction of the WOLK hip airbag, the occurrence of hip and pelvic fractures declined by 45% whereas the number of falls only declined by 12%. These results may indicate that in selected older patients residing in long-term care facilities, a hip airbag can be used to lower the number of hip and pelvic fractures. However, a randomized clinical trial is needed to verify these results.

A main strength of our study is the before and after comparison following an intervention in a controlled environment. All participating long-term care facilities had similar care protocols throughout the study periods. Admission criteria remained stable, meaning that patients with the same indications were admitted to the long-term care facilities during the study. This lowers the risk of confounding by different treatment protocols or treatment variation over time. Furthermore, only 45 WOLK hip airbags were introduced among > 900 individuals, indicating that selection of patients at highest risk of falling contributed strongly to the effectiveness. The hip airbag was mainly used, but not restricted to, individuals with wandering behaviour. It is therefore likely that, with only 45 hip airbags, the estimated incidence rate ratios might be an underestimation of the true effect, in case more or even all individuals would have worn a device. The findings of this study are strengthened by the fact that the incidence of other fractures such as lower-leg or humeral fractures did not decline following hip airbag application, rendering it unlikely that increased awareness of falling and other measures to reduce falling are mainly responsible for the effect demonstrated in this study.

Unfortunately, as data were collected on a population level (and not individually due the pilot study design) we were not able to study the effect of the hip airbag in the subgroup of patients who used the hip airbag. The lack of data on an individual patient level is the main limitation of this study. As such, patient characteristics between study periods may have differed on factors such as changes in frailty, cognitive impairment, behavioural symptoms of dementia, medication use or reduced physical function. This could have induced confounding (leading to a reduced incidence of falling) which we were not able to adjust for. A minor (12%) but significant reduction in falls over the study period (IRR 0.88, 95% CI 0.83–0.93) was found which might suggest that pre-and post-intervention patient characteristics were not completely similar. Although all long-term care facilities and admission criteria remained equal throughout the study, we can’t rule out that a part of the reduced risk on hip fractures was introduced by confounding. This would imply that the effectiveness of the hip-airbag was overestimated. Second, due to the nature of this study, using electronic care data from the long-term care facility, underreporting of fractures could be an issue. Yet, the incidence of hip fractures in our study (mean 2.5/100 person years) is similar to previous studies of similar nature. In a dataset among 1.4 million persons residing in United States nursing homes identified by Medicare data, the incidence of hip fractures was 2.3/100 persons years, 74% were women, as compared to 70% in our study [17]. Another study aimed to investigate the effectiveness of hip protectors among residents in long-term care facilities. In this study the incidence of hip fractures was 0.92/100 falls in persons without a hip protector. Our study showed that 1.36 hip fractures per 100 falls occurred in the control period. These numbers do not hint towards an underreported incidence in our study and thus a reliable fracture registry was present.

Earlier studies focusing on hip protectors failed to convincingly show a reduced effect on hip fractures in residents in long-term care facilities. In a Cochrane review, including 11.808 participants, a small risk reduction for hip fractures was observed (RR 0.82 (95%CI 0.67–1.00). Of note, in most of these trials, the intervention:control ratio was 1:1, indicating that at least 50% of the study population did receive a hip protector. In our study, only 45 hip airbags were deployed and despite that, a much stronger effect was found (RR 0.56 (95%CI 0.34–0.92) in the complete population. From all included studies in the Cochrane, compliance rates ranged between 37% and 72% (median 68%) [14]. In our study, compliance was not studied; in case a participant refused to wear the hip airbag, it was used to help another participants. As only 45 WOLK hip airbags were used among 969 individuals it is unlikely that difference in compliance led to the differences in effectiveness. By design, the compliance in our study was only 45/969 (= 5%) at most.

The incidence of hip fractures in our study was of similar nature as compared to previous trials that studied the effect of hip protectors. In our study, in the control period, the incidence of hip fractures was 2.9/100py. Earlier studies showed an incidence for hip fractures of 1.2/100py [18], 1.3/100py [19], 1.7/100py [20], 2.1/100py [21], 2.5/100py [16], 3.7/100py [22], 4.6/100py [23]. The similar incidence found in previous studies reassures that our study population was not a selected “high risk” population. Additionally, no hip fractures occurred during a fall in participants wearing the WOLK hip airbag during that fall. This strengthens our findings that almost 50% of hip fractures were prevented by only 45 hip airbags among 969 participants. In contrast, in all previous randomized controlled trials studying the effect of a hip protector, hip fractures did occur during a fall when the hip protector was worn [13].

The findings of the study may have implications for clinical care in the older patients. For individuals in long-term care facilities, wearing a hip airbag could potentially reduce the number of hip and pelvic fractures in those who frequently fall. However, the results of this study warrant a future randomized clinical trial to verify these results and to determine true effectiveness of the intervention. Before such a clinical trial is performed, statements on the effectiveness are uncertain. Even despite the fact that the effect of the hip airbag on prevention of hip and pelvic fractures is probably underestimated as the number of hip airbags was insufficient to treat the whole study population. Reduction of hip and pelvic fractures inevitably leads to less mortality or loss of independence. Besides hip fracture reduction, other indirect effects were also noticed during the study; there was more time to care for patients (instead of paying attention to control those with high risk of falling). Of note, the effects on mortality, loss of independence and other indirect effect were not measured during this pilot study and should be included in a future clinical trial.

Although this study focused on application of a hip airbag in an inpatient setting, the results of this study are also of interest to apply on outpatients, i.e. older patients who fall frequently at home. In this case, the hip airbag could potentially lead to prolonged independence at home as hip or pelvic fractures may be prevented here as well. This again, would also be of interest for a future clinical trial.

## Conclusions

In this pilot study the first results of application of a hip airbag were reported. Among permanent residents of long-term care facilities, the incidence of hip and pelvic fractures was reduced with 45%. However, as this pilot study may have been subject to bias, effectiveness of the hip airbag needs to be studied in a subsequent replication study, preferably a randomized clinical trial. Such a trial should focus on hip airbag effectiveness, both in an inpatient as outpatient setting. In addition to effectiveness, compliance, mortality, loss of independence (in an outpatient setting) and cost-effectiveness of hip airbag application are of interest in a future clinical trial.

## Data Availability

The data that support the findings of this study are not publicly available as these might contain information that could compromise the privacy of research participants. However, data will be available (conditional on agreement on privacy matters and appropriate usage of the data) upon request at tanteLouise, science practitioner: Katja Drost.

## Abbreviations

CI: Confidence Interval
IR: Incidence Rate
IRR: Incidence Rate Ratio
RR: Relative Risk
PY: Patient Year
