# Correction: Prevention of hip fractures in older adults residing in long-term care facilities with a hip airbag: a retrospective pilot study

**DOI:** 10.1186/s12877-022-03267-1

**Published:** 2022-07-27

**Authors:** Banne Nemeth, Marleen van der Kaaij, Rob Nelissen, Jan-Kees van Wijnen, Katja Drost, Gerard Jan Blauw

**Affiliations:** 1 Department of Clinical Epidemiology, Leiden University Medical Center, Albinusdreef 2, Leiden, 2333 ZA The Netherlands; 2 Department of Orthopedic Surgery, Leiden University Medical Center, Albinusdreef 2, Leiden, 2333 ZA The Netherlands; 3 Department of Internal Medicine, Geriatrics, Amstelland Ziekenhuis, Amstelveen, The Netherlands; 4 tanteLouise, Bergen op Zoom, The Netherlands; 5 Department of Internal Medicine, Geriatrics, Leiden University Medical Center, Leiden, The Netherlands

**Correction: BMC Geriatr 22, 547 (2022)**

**https://doi.org/10.1186/s12877-022-03221-1**

After publication of this article [1], the authors reported that the wrong figure appeared as Fig. 2; the figure should have appeared as shown below.

**Fig. 2:**
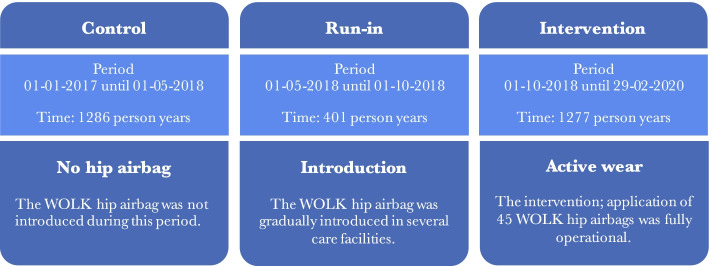
Study period overview

The original article [1] has been corrected.
